# Contrast media timing optimization for coronary CT angiography: a retrospective validation study in swine

**DOI:** 10.1007/s00330-022-09161-z

**Published:** 2022-10-11

**Authors:** Logan Hubbard, Shant Malkasian, Yixiao Zhao, Pablo Abbona, Sabee Molloi

**Affiliations:** Department of Radiological Sciences, Medical Sciences I, B-140, University of California - Irvine, Irvine, CA 92697 USA

**Keywords:** Coronary artery disease, Computed tomography angiography, Contrast media

## Abstract

**Objectives:**

The objective was to retrospectively develop a protocol in swine for optimal contrast media timing in coronary CT angiography (CCTA).

**Methods:**

Several dynamic acquisitions were performed in 28 swine (55 ± 24 kg) with cardiac outputs between 1.5 and 5.5 L/min, for 80 total acquisitions. The contrast was injected (1mL/kg, 5mL/s, Isovue 370), followed by dynamic scanning of the entire aortic enhancement curve, from which the true peak time and aortic and coronary enhancements were recorded as the reference standard. Each dataset was then used to simulate two different CCTA protocols—a new optimal protocol and a standard clinical protocol. For the optimal protocol, the CCTA was acquired after bolus tracking-based trigging using a variable time delay of one-half the contrast injection time interval plus 1.5 s. For the standard protocol, the CCTA was acquired after bolus tracking-based triggering using a fixed time delay of 5 s. For both protocols, the CCTA time, aortic enhancement, coronary enhancement, and coronary contrast-to-noise ratio (CNR) were quantitatively compared to the reference standard measurements.

**Results:**

For the optimal protocol, the angiogram was acquired within −0.15 ± 0.75 s of the true peak time, for a mean coronary CNR within 7% of the peak coronary CNR. Conversely, for the standard CCTA protocol, the angiogram was acquired within −1.82 ± 1.71 s of the true peak time, for a mean coronary CNR that was 23% lower than the peak coronary CNR.

**Conclusions:**

The optimal CCTA protocol improves contrast media timing and coronary CNR by acquiring the angiogram at the true aortic root peak time.

**Key Points:**

• *This study in swine retrospectively developed the mathematical basis of an improved approach for optimal contrast media timing in CCTA.*

• *By combining dynamic bolus tracking with a simple contrast injection timing relation, CCTA can be acquired at the peak of the aortic root enhancement.*

• *CCTA acquisition at the peak of the aortic root enhancement should maximize the coronary enhancement and CNR, potentially improving the accuracy of CT-based assessment of coronary artery disease.*

**Supplementary Information:**

The online version contains supplementary material available at 10.1007/s00330-022-09161-z.

## Introduction

Coronary computed tomography angiography (CCTA) is a powerful tool for noninvasive assessment of coronary artery disease, where plaque burden and stenosis severity are highly predictive of major adverse cardiac events [1]. In particular, CCTA is the only non-invasive modality with class 1, level A evidence for assessment of acute or stable chest pain [2], where patient outcomes are improved as compared to standard care alone [3]. Nevertheless, CCTA quality is affected by many factors, including motion, multi-beat phase misregistration, blooming, beam hardening, and contrast timing [4].

Regarding contrast timing, the accuracy of CCTA depends directly upon the coronary enhancement [5, 6]. While contrast media concentration and volume can be increased to boost enhancement, CCTA acquisition at the peak enhancement of the aortic root yields the highest intracoronary enhancement [7, 8]. Presently, two strategies are used for CCTA acquisition timing: test bolus and bolus tracking. The former employs a small contrast injection (10–15 mL), where the resulting cardiac output–dependent time-attenuation curve is used to estimate both the bolus arrival and peak acquisition time [6]. Nevertheless, the time-to-peak predictions often vary in practice [9] while requiring extra contrast media, radiation dose, and exam time. Alternatively, for bolus tracking, contrast media is injected, a region-of-interest is dynamically tracked over time, and upon reaching a particular blood pool enhancement (typically 100–200 Hounsfield units (HU)), triggering occurs with CCTA acquisition after a fixed delay [10]. Hence, bolus tracking can also account for cardiac output–dependent differences in bolus arrival time. Yet, it relies on a fixed acquisition delay following triggering, i.e., the patient-specific delay time necessary for optimal acquisition at the true aortic contrast bolus peak remains unknown.

Thus, the objective of this study was to retrospectively develop a CCTA protocol in swine for optimal contrast media timing with improved coronary enhancement, where a standard clinical CCTA protocol was also employed for comparison The optimal protocol combines 2-mm slice dynamic bolus tracking with a variable acquisition delay following triggering, where this delay—equal to one-half the contrast injection time interval plus a fixed dispersion time [11–14]—enables reliable CCTA acquisition at the aortic root peak time.

## Materials and methods

### General methods

The study was approved by the Institutional Animal Care and Use Committee and was performed using data that were previously acquired from 28 male Yorkshire swine (55 ± 24 kg) [14], where the present work is new and independent. The study was organized into three parts: (1) data acquisition, (2) modeling, and (3) protocol simulation.

- Data acquisition: Several dynamic acquisitions were conducted per animal under rest and stress conditions, as previously reported [14] and discussed below, with cardiac outputs ranging from 1.5–5.5 L/min.
- Modelling: The aortic contrast bolus geometry of each acquisition was characterized using automatic gamma variate fitting, where the true aortic peak time, aortic enhancement, coronary enhancement, and coronary contrast-to-noise ratio (CNR) were recorded as the reference standard.
- Protocol simulation: The model fit curves of each acquisition were used retrospectively to simulate the optimal CCTA protocol as well as a standard clinical CCTA protocol for comparison. For each protocol, the CCTA acquisition time, aortic enhancement, coronary enhancement, and coronary CNR were compared to the reference standard measurements.

### Data acquisition

#### Animal preparation

Induction of anesthesia was achieved with Telazol (4.4 mg/kg), Ketamine (2.2 mg/kg), and Xylazine (2.2 mg/kg), and was maintained with 1.5–2.5% Isoflurane (Highland Medical Equipment and Baxter) [14]. Introducer sheaths were placed (5 Fr, AVANTI^®^, Cordis Corporation) in each femoral vein and were used for contrast media injection, intravenous fluids, and adenosine administration.

#### Imaging and reconstruction

**Fig. 1:**
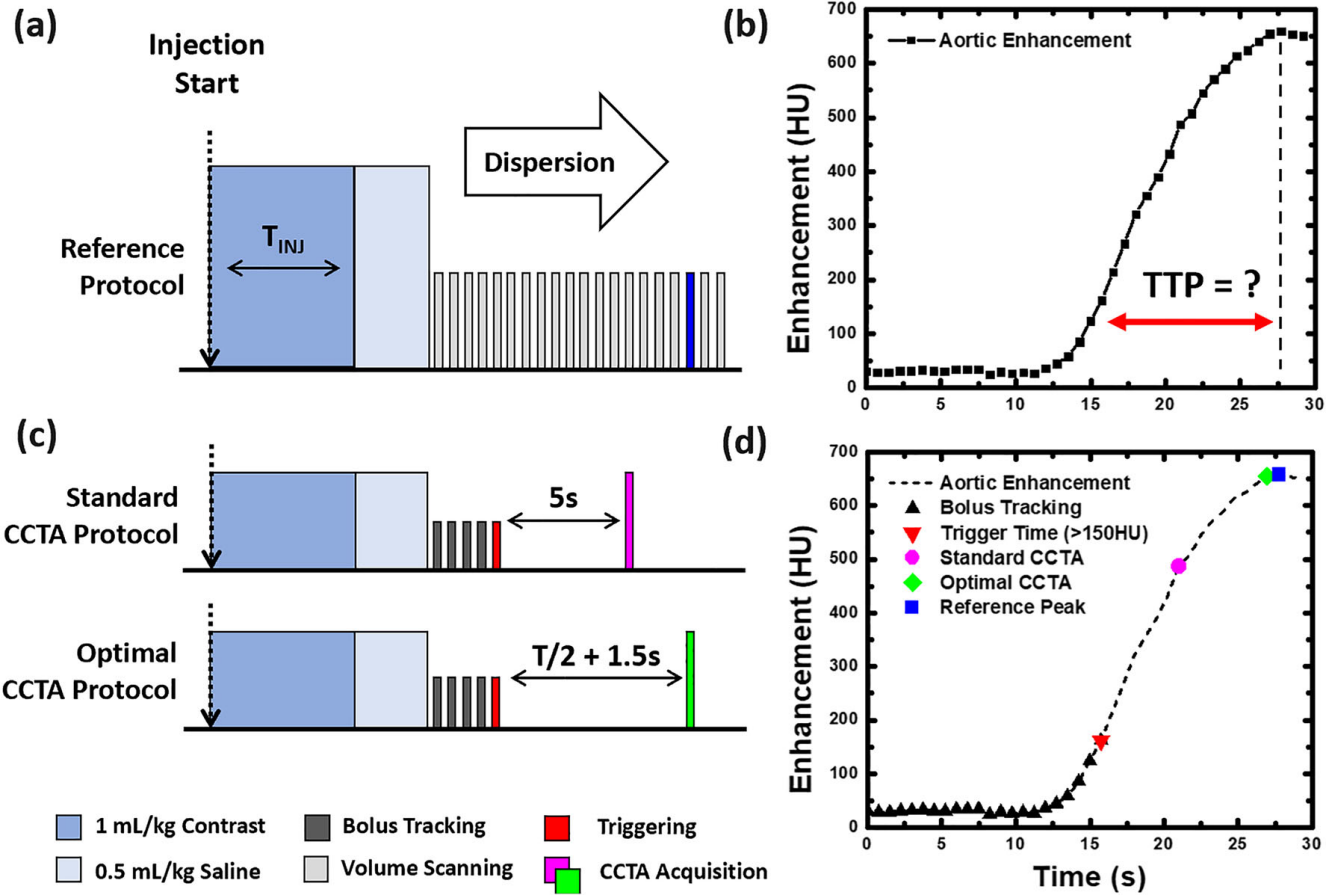
Bolus dispersion, reference standard, and CCTA protocol simulation. **a** During injection, the contrast bolus has a fixed concentration per unit time at the venous injection site. Following mixing and dispersion, reference imaging reveals (**b**) an approximately gamma variate bolus geometry, with a width proportional to the contrast injection time (T_INJ_). Using the reference data, the peak time and enhancement were recorded, while the optimal trigger-to-peak (TTP) time was unknown. **c** The standard CCTA and optimal CCTA protocols were then simulated, where dynamic bolus tracking with triggering at 150 HU was performed, after which time delays of 5 s and T_INJ_ / 2 + 1.5 s were used, respectively, for CCTA acquisition. **d** An example case of bolus tracking, triggering, standard CCTA, and optimal CCTA as compared to the reference peak of the contrast bolus are shown

For the 28 animals assessed, approximately half underwent one rest and one stress acquisition (for a total of two acquisitions), while the other half underwent two rest and two stress acquisitions (for a total of four acquisitions), where stress was used (240 μg adenosine/kg/min, Model 55-2222, Harvard Apparatus) to modulate cardiac output [14]. For each acquisition, 1 mL/kg of contrast material (Isovue 370, Bracco Diagnostics) was injected at 5 mL/s (Empower CTA, Acist Medical Systems) for an iodine delivery rate of 1850 mg/s, followed by a 0.5 mL/kg saline chaser. Dynamic scanning was then performed at 100 kVp and 200 mA over 20–30 s (Aquilion One, Canon Medical Systems) to capture the entire aortic enhancement curve, as shown in Fig. 1a and b. After which, a 10-min delay was observed between consecutive acquisitions. All scans were acquired as full projection data with a rotation time of 0.35 s, a collimation of 320 x 0.5 mm, and a cranio-caudal coverage of 16 cm with zero detector pitch. All reconstructions were performed at 75% of the R-R interval using AIDR 3D, an FC03 kernel, and a voxel size of 0.43 × 0.43 × 0.5 mm.

### Enhancement curve modeling

For each acquisition series, the central lumen of the aortic root was segmented semi-automatically (Vitrea fX version 6.0, Vital Images, Inc.), to yield a 5 mL vascular volume-of-interest (VOI). This VOI was used to generate an enhancement curve that was automatically fit with a gamma variate function (LSQCurveFit, MatLab 2013a, MathWorks) [13, 14], as shown in Eq. 1.

1 $$ \mathrm{Aortic}\kern.3em \mathrm{enhancement}\kern.3em \left(\boldsymbol{t}\right)=\boldsymbol{A}\times {\left(\frac{\boldsymbol{t}}{\boldsymbol{\tau}}\right)}^{\boldsymbol{B}}\times {\exp}^{\boldsymbol{B}\times \left(\mathbf{1}-\frac{\boldsymbol{t}}{\boldsymbol{\tau}}\right)}+\boldsymbol{C} $$

*A* is the maximum enhancement, *t* is time, *τ* is the peak time, *B* is the growth factor, and *C* is the initial pre-contrast blood pool enhancement. The resulting fit curves were then used to identify the volume scan at the aortic root peak, after which the reference aortic root enhancement, coronary enhancement, and coronary CNR were determined. Specifically, the aortic VOI was used to measure the mean and standard deviation of the aortic root enhancement. Next, volumetric segments of the proximal left main (LM) and right coronary (RCA) arterial lumens were segmented semiautomatically (Vitrea fX version 6.0, Vital Images, Inc.), to measure the mean coronary enhancements. Finally, the CNR of the LM and RCA were calculated as the mean coronary enhancement minus the surrounding tissue enhancement normalized by the standard deviation of the aortic root enhancement. Finally, each aortic fit curve was used to simulate the optimal and standard CCTA protocols.

### CCTA protocol simulations

#### Optimal CCTA protocol

During contrast injection, the bolus enters the venous blood pool at a high rate, remaining largely unmixed over the injection duration, as displayed in Fig. 1a. Following injection, however, the bolus travels through the venous circulation, right cardiac chambers, pulmonary circulation, and left cardiac chambers, where significant mixing and dispersion occur. The result at the aortic root is an approximately gamma variate bolus geometry, where the bolus’ temporal width is proportional to the volume of contrast injected [11, 12]. Assuming a fixed injection rate, it follows that the bolus width is also proportional to the bolus injection time [13, 14], where one-half the injection time ($\frac{T_{INJ}}{2}$) plus a fixed dispersion time (*T*_*d*_) corresponds to the trigger-to-peak enhancement time of the bolus (*T*_*TTP*_), as shown in Eq. 2.

2 $$ {\boldsymbol{T}}_{\boldsymbol{T}\boldsymbol{TP}}=\frac{{\boldsymbol{T}}_{\boldsymbol{INJ}}}{\mathbf{2}}+\boldsymbol{Td} $$

Hence, the optimal CCTA protocol applied this peak timing theory to the aortic fit curves. First, 2-mm slice dynamic bolus tracking was simulated with triggering at 150 HU in the aortic root to emulate clinical practice. Following triggering, a variable time delay of one-half the contrast injection duration plus a dispersion delay of 1.5 s was employed for all animals, after which the first available ECG-gated volume scan was “acquired” as the CCTA, as shown in Fig. 1c. The acquisition time was then recorded, and the aortic root enhancement, LM and RCA enhancements, and CNRs were computed.

#### Standard CCTA protocol

Using the same aortic fit curves, 2-mm slice dynamic bolus tracking was again simulated with triggering at 150 HU in the aortic root as above. Following triggering, a fixed time delay of 5 s was employed, after which the first available ECG-gated volume scan was “acquired” as the standard clinical CCTA, as shown in Fig. 1c. The acquisition time was then recorded, and the aortic root enhancement, LM and RCA enhancements, and CNRs were computed.

### Statistical approach

As our prior work found no significant differences in bolus time-to-peak between rest and stress [14], all rest and stress data were pooled for analysis. First, the mean trigger-to-acquisition time, aortic enhancement, coronary enhancement, and coronary CNR of both the optimal and standard CCTA protocols were compared to the reference standard measurements through paired sample t-testing (*p* < 0.05), where the errors of such measurements were also computed. Second, the trigger-to-acquisition time and aortic enhancement of both the optimal and standard CCTA protocols were compared to the reference standard through regression, where Pearson’s correlation (r) and root-mean-square-error (RMSE) of measurement were also determined. Finally, the trigger-to-acquisition time error and aortic enhancement error of both the optimal and standard CCTA protocols as a function of contrast injection time were assessed through regression, where Pearson’s correlation (r) and root-mean-square-error (RMSE) of measurement were also computed. Statistical software was used for all analyses (MatLab 2013a, MathWorks; SPSS, Version 22, IBM Corporation).

## Results

### General

A total of 80 acquisitions were completed in the 28 swine with cardiac outputs ranging from 1.5 to 5.5 L/min. The weight-based contrast media injection volumes ranged from 27 to 95 mL, corresponding to the 55 ± 24 kg weights of the animals, while the contrast media injection times, excluding the saline chaser, ranged from 5.4 to 19.0 s (given the fixed 5 mL/s rate of injection).

### Mean comparisons

The mean trigger-to-acquisition times of the optimal and standard CCTA protocols were 7.04 ± 1.49 and 5.37 ± 0.21 s, respectively, while the corresponding trigger-to-reference peak time was 7.19 ± 1.68 s. Moreover, the mean aortic enhancements of the optimal and standard CCTA protocols were 770.54 ± 135.30 and 727.31 ± 143.12 HU, respectively, while the corresponding reference aortic enhancement was 787.07 ± 142.39 HU. Likewise, the mean combined coronary enhancements (LM and RCA) of the optimal and standard CCTA protocols were 455.64 ± 182.34 and 421.39 ± 170.10 HU, respectively, while the corresponding reference coronary enhancement was 487.57 ± 179.52. Finally, the mean combined coronary CNR (LM and RCA) of the optimal and standard CCTA protocols were 9.69 ± 6.62 and 8.18 ± 6.09 HU: approximately 7% and 23% lower than the corresponding reference CNR of 10.64 ± 6.41, respectively. The above data, individual coronary data, and the corresponding t-testing and error analysis are detailed in Table 1.

**Table 1:** CCTA protocol timing, enhancement, and CNR comparisons versus the reference standard

PROTOCOL (*N* = 80)	Parameter (Mean ± SD)	Reference (Mean ± SD)	Error (Mean ± SD)	*p* value (α < 0.05)
Standard CCTA (+5 s)				
*Trigger-to-acquisition time (s)*	5.37 ± 0.21	7.19 ± 1.68	–1.82 ± 1.71	0.00**
*Aortic enhancement (HU)*	727.31 ± 143.12	787.07 ± 142.39	–59.76 ± 48.99	0.05
*LM enhancement (HU)*	425.38 ± 189.42	509.80 ± 179.69	–84.41 ± 83.52	0.04**
*RCA Enhancement (HU)*	417.40 ± 151.40	465.35 ± 179.51	–47.94 ± 60.49	0.13
*LM and RCA enhancement (HU)*	421.39 ± 170.10	487.57 ± 179.52	–66.18 ± 74.62	0.02**
*LM CNR*	8.35 ± 6.54	11.39 ± 6.23	–3.04 ± 3.13	0.03**
*RCA CNR*	8.01 ± 5.71	9.90 ± 6.61	–1.88 ± 2.05	0.12
*LM and RCA CNR*	8.18 ± 6.09	10.64 ± 6.41	–2.46 ± 2.69	0.02**
OPTIMAL CCTA (+T_INJ_ / 2 + 1.5 s)				
*Trigger-to-acquisition time (s)*	7.04 ± 1.49	7.19 ± 1.68	–0.15 ± 0.75	0.07
*Aortic enhancement (HU)*	770.54 ± 135.30	787.07 ± 142.39	–16.53 ± 19.82	0.32
*LM enhancement (HU)*	475.12 ± 181.33	509.80 ± 179.69	–34.68 ± 78.27	0.22
*RCA enhancement (HU)*	436.16 ± 184.23	465.35 ± 179.51	–29.18 ± 52.98	0.27
*LM and RCA enhancement (HU)*	455.64 ± 182.34	487.57 ± 179.52	–31.93 ± 66.34	0.16
*LM CNR*	10.38 ± 6.66	11.39 ± 6.23	–1.01 ± 2.57	0.27
*RCA CNR*	8.99 ± 6.62	9.90 ± 6.61	–0.90 ± 1.79	0.30
*LM and RCA CNR*	9.69 ± 6.62	10.64 ± 6.41	–0.96 ± 2.20	0.21

*CCTA*, coronary computed tomography angiography; *N*, number of measurements; *HU*, Hounsfield units; *Standard*, standard CCTA protocol that uses a fixed delay of 5 s; *Optimal*, optimal CCTA protocol that uses a variable delay of ½* total injection time + 1.5 s; *LM*, left main coronary artery; *RCA*, right coronary artery; *CNR*, contrast-to-noise ratio. *Reference*, corresponding result of the reference standard peak time or enhancement. **Indicates a *p* value less than 0.05, i.e., significantly different from the reference standard

### Regression analysis

**Fig. 2:**
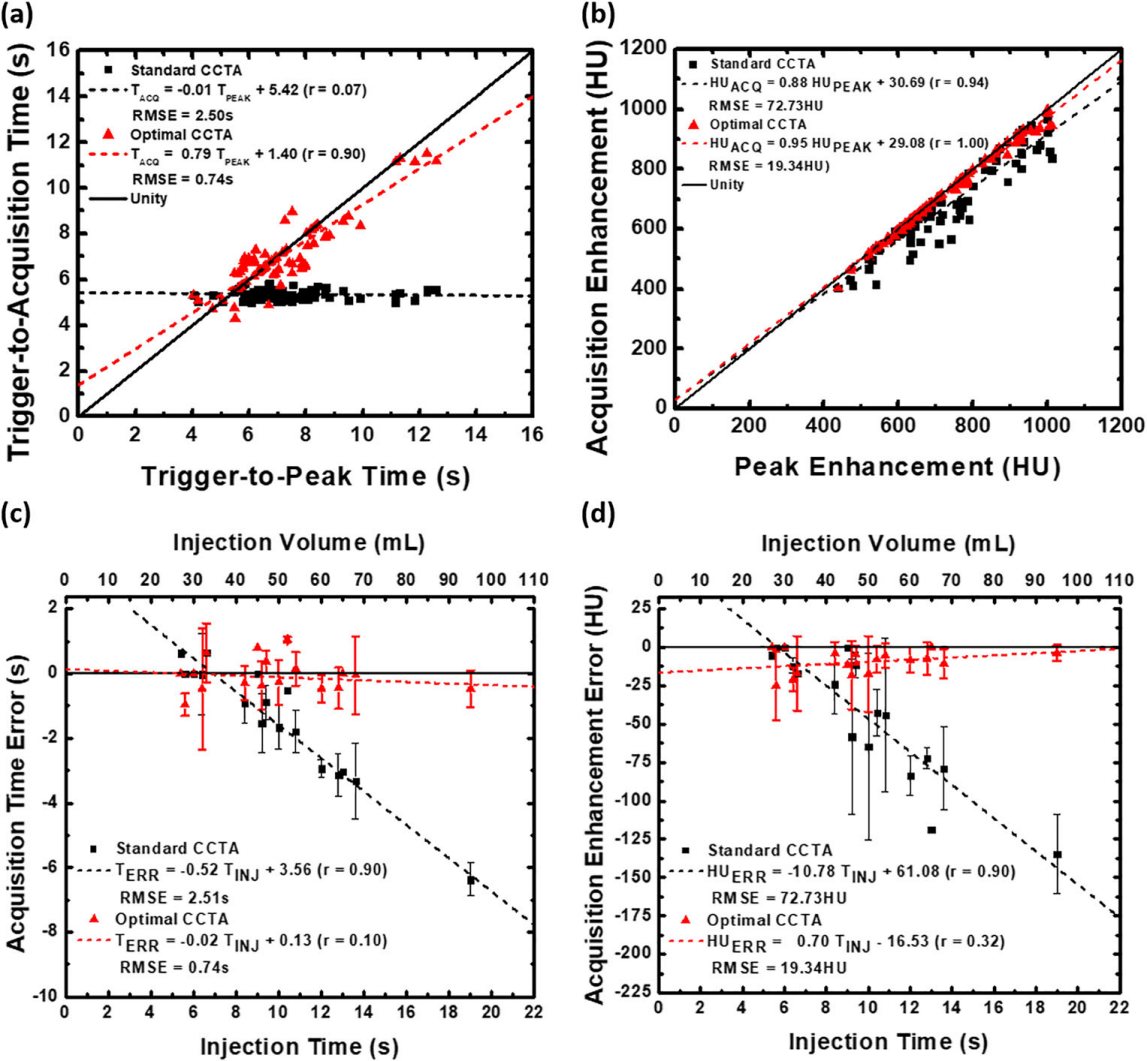
Acquisition timing and aortic enhancement of the standard and optimal CCTA protocols as compared to the reference standard. **a** Protocol-dependent trigger-to-acquisition time versus the reference trigger-to-peak time. **b** Protocol-dependent aortic enhancement versus the reference peak aortic enhancement. **c** Protocol-dependent acquisition time error relative to the reference peak time as a function of injection time (or injection volume, as displayed by the double axis). **d** Protocol-dependent aortic enhancement error relative to the reference peak aortic enhancement as a function of injection time (or injection volume, as displayed by the double axis). In all cases, the standard CCTA protocol data is shown in black, with the optimal CCTA protocol data is shown in red. Importantly, for panels c and d, clinically realistic injection times and volumes range from 10 or more seconds and 50–120 mL, respectively, at a rate of 5–7 mL/s [15]

When comparing the trigger-to-acquisition time (*T*_ACQ_) of the optimal CCTA protocol to the trigger-to-reference peak time (*T*_PEAK_), the data were related through regression by *T*_ACQ_ = 0.79 *T*_PEAK_ + 1.40, with a Pearson’s correlation of 0.90 and a RMSE of 0.74 s. When comparing the trigger-to-acquisition time (*T*_ACQ_) of the standard CCTA protocol to the trigger-to-reference peak time (*T*_PEAK_), the data were related through regression by *T*_ACQ_ = −0.01 *T*_PEAK_ + 5.42, with a Pearson’s correlation of 0.07 and a RMSE of 2.50 s, as shown in Fig. 2a. Additionally, when comparing the aortic enhancement (HU_ACQ_) of the optimal CCTA protocol to the reference aortic enhancement (HU_PEAK_), the data were related through regression by HU_ACQ_ = 0.95 HU_PEAK_ + 29.08, with a Pearson’s correlation of 1.00 and RMSE of 19.34 HU. When comparing the aortic enhancement (HU_ACQ_) of the standard CCTA protocol to the reference aortic enhancement (HU_PEAK_), the data were related through regression by HU_ACQ_ = 0.88 HU_PEAK_ + 30.69, with a Pearson’s correlation of 0.94 and a RMSE or 72.73 HU, as shown in Fig. 2b.

### Error analysis

**Fig. 3:**
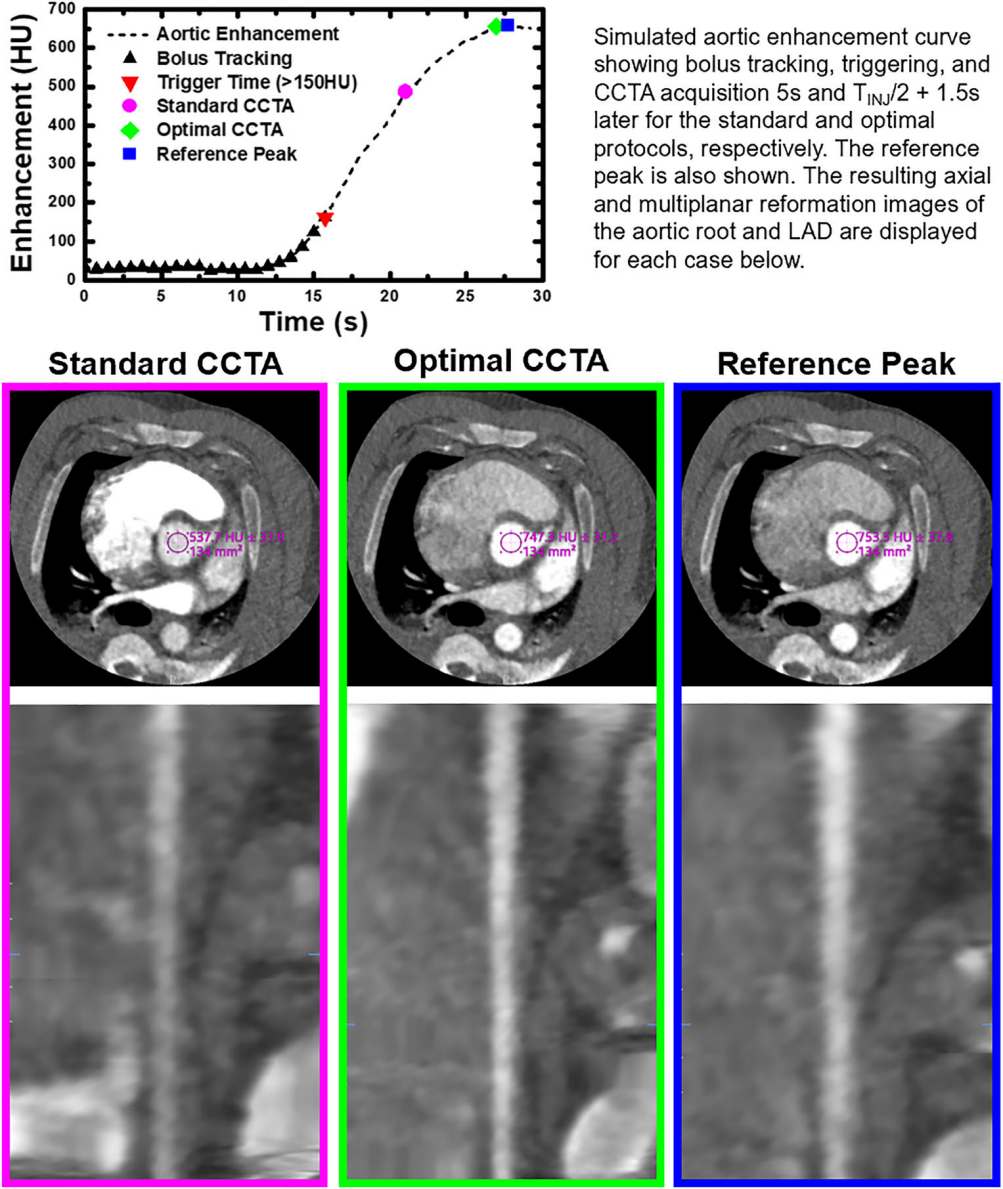
Qualitative assessment of the standard and optimal CCTA aortic and coronary enhancement as compared to the reference aortic and coronary enhancement. An example case is shown from a 95 kg animal, where the standard CCTA protocol underestimates the peak timing, while the optimal CCTA protocol accurately predicts the peak timing. Axial and multiplanar reformation images of the aortic root and left anterior descending coronary artery are shown in each case versus the corresponding reference data

When assessing the trigger-to-acquisition time error (*T*_ERR_) of the optimal CCTA protocol as a function of contrast injection time (*T*_INJ_), the data were related through regression by *T*_ERR_ = −0.02 T_INJ_ + 0.13, with a Pearson’s correlation of 0.10 and a RMSE of 0.74 s. When assessing the trigger-to-acquisition time error (*T*_ERR_) of the standard CCTA protocol as a function of contrast injection time (*T*_INJ_), the data were related through regression by *T*_ERR_ = −0.52 T_INJ_ + 3.56, with a Pearson’s correlation of 0.90 and a RMSE of 2.51 s, as shown in Fig. 2c. When assessing the aortic enhancement error (HU_ERR_) of the optimal CCTA protocol as a function of contrast injection time (*T*_*I*NJ_), the data were related through regression by HU_ERR_ = 0.70 *T*_INJ_ −16.53, with a Pearson’s correlation of 0.32 and a RMSE of 19.34 HU. When assessing the aortic enhancement error (HU_ERR_) of the standard CCTA protocol as a function of contrast injection time (*T*_INJ_) the data were related through regression by HU_ERR_ = −10.78 T_INJ_ + 61.08, with a Pearson’s correlation of 0.90 and a RMSE of 72.73 HU, as shown in Fig. 2d. Overall, to visualize this aortic enhancement error as well as its impact on coronary enhancement, an example case of the standard and optimal CCTA protocols implemented in a 95kg animal is shown in Fig. 3. Notably, a 95 kg animal was used as an example to emulate a realistic patient weight, contrast volume, and injection time as is used clinically.

### Supplemental Data

Since CCTA triggering on volumetric scanners is sometimes performed using the descending aorta, the same modelling, delay, and simulation were used to assess the optimal CCTA protocol with the descending aorta as the bolus tracking trigger site, where the aortic root remained the reference standard. Corresponding mean comparisons, regression, and error analyses are shown in Supplemental Table 1 and Supplemental Figure 1.

## Discussion

### Indication of results

For the optimal CCTA protocol, reliable CCTA timing at or near the true peak of the aortic enhancement improved coronary CNR, where such reliability was maintained with minimal timing and enhancement error, over a clinically realistic range of cardiac outputs, injection times, and volumes [15]. Hence, one-half the contrast injection duration plus a fixed dispersion time was strongly predictive of the true aortic peak time following triggering, regardless of animal weight. Conversely, the standard CCTA protocol could not reliably time the angiogram at or near the true peak of the aortic bolus, where timing and enhancement errors increased proportionally with injection time and volume.

### Comparison to previous work

Optimizing coronary enhancement and CNR is of major clinical importance, not only for improved coronary artery disease assessment but also for more efficacious use of contrast material. Elements that influence coronary enhancement and CNR include CT scanning parameters, contrast concentration, and injection rate, i.e., the iodine delivery rate, contrast volume, circulating blood volume, and cardiac output, where the latter four were the primary focuses of this work. Regarding iodine delivery rate, contrast volume, and circulating blood volume, CCTA protocols commonly employ 320–370 mg/mL concentration contrast at injection rates of 5mL/s or more with fixed injection volumes ranging from 50 to 120 mL [15], regardless of circulating blood volume. However, for any bolus of contrast injected, the degree of arterial enhancement correlates inversely with the circulating blood volume, which scales approximately with body weight or body surface area (albeit with some deviation at extremes of mass) [16, 17]. Hence, the total volume of contrast injected for CCTA should be patient-specific, where larger patients (with larger blood volumes) should receive larger volumes of contrast for an optimal coronary enhancement between 325 and 500 HU [5, 6, 15, 18]. As such, the present study employed a simple weight-based injection protocol (1mL of 370 mg/mL contrast per kg body mass) at a high rate of 5 mL/s to yield an average coronary enhancement of ~450 HU across all 28 animals assessed. Importantly, however, swine have a smaller volume of distribution as compared to humans; hence, the contrast volume or iodine delivery rate used clinically will need to be adjusted accordingly to achieve a particular coronary enhancement.

Additionally, it is known that bolus arrival time and max aortic peak enhancement are inversely related to cardiac output [19, 20]. Hence, dynamic bolus tracking with near real-time slice reconstruction and aortic enhancement monitoring provides a convenient solution for triggering as it accounts for patient-specific differences in bolus arrival time, where dual triggering can further improve reliability [21, 22]. Following triggering, however, the optimal time delay for acquisition at the true peak of the aortic root enhancement remains unknown. Fortunately, models have been developed to better characterize this optimal delay using bolus kinetic and transfer function modelling [23–26], but in practice, such models are not widely used as they require several steps and data inputs. Nevertheless, work by Hinzpeter et al and Korporaal et al showed improved aortic enhancement when employing patient-specific variable time delays versus fixed trigger delays, where the patient-specific delays are determined via reconciliation of bolus tracking data with a large-scale database of known arterial enhancement curves [26, 27]. Yet, Korporaal et al also reported the patient-specific delays between triggering and peak acquisition increased linearly with contrast injection duration [27]. Hence, our optimal timing protocol agrees with the findings of Hinzpeter et al and Korporaal et al, but it is fundamentally simpler.

As such, the results of the present study represent a new approach to contrast media timing in CCTA for optimal coronary enhancement and CNR. First, an acquisition delay of one-half the contrast injection time plus a fixed dispersion time was used to scale proportionally with the contrast injection time, as bolus width is a function of contrast injection time [11, 12, 20, 21]. Second, bolus tracking was used to account for differences in cardiac output–dependent bolus arrival times. And third, a weight-based volume of contrast was injected to scale with central blood volume and maintain coronary enhancement. In combination, the optimal CCTA protocol represents a simple yet robust solution to improve contrast media timing, coronary enhancement, and CNR.

### Limitations

Despite the advantages of the optimal CCTA protocol, the present work has several limitations. Most importantly, the optimal CCTA protocol was validated retrospectively using temporally oversampled datasets and simulation. While the use of simulation did enable the protocol to be developed and compared directly to the reference standard without confounding factors, the performance of the technique in real-time during a breath-hold was not assessed. Hence, prospective implementation of the optimal CCTA protocol versus a standard CCTA protocol remains an important and necessary next step. Similarly, the optimal CCTA protocol was validated using volumetric data from a wide-detector CT scanner without couch motion. Thus, for CT scanners with smaller Z-axis detector coverage, the use of spiral or shuttle-mode acquisitions is needed where the optimal time delay after triggering will need to be shortened by one-half the spiral or shuttle mode acquisition duration. Another limitation of this work was that it was performed in swine, weighing 52 ± 15 kg, without significant disease, using the femoral vein as the injection site. While clinically realistic injection volumes were used in most cases at an injection rate of 5mL/s, with cardiac outputs ranging from 1.5 to 5.5 L/min (owing to the use of adenosine), future work should still employ larger animals with significant disease using a more peripheral injection site, where different injection volumes and rates should be assessed for more robust validation of the protocol. Finally, future work should employ half-projection scanning or reconstruction to minimize gantry-rotation-induced motion artifacts, while trained readers could also be used for quality assessment scoring [28].

### Conclusion

This study in swine retrospectively developed the mathematical basis of an improved approach for optimal contrast media timing in CCTA. Specifically, by combining dynamic bolus tracking with a simple contrast injection timing relation, reliable timing of the angiogram at the true peak of the aortic root enhancement is feasible. As a result, coronary enhancement and contrast-to-noise ratio should be maximized, potentially improving the accuracy of CT-based assessment of coronary artery disease.

## Supplementary information

ESM 1 (DOCX 492 kb)

## Abbreviations

CCTA: Coronary computed tomography angiography
CNR: Contrast-to-noise ratio
HU: Hounsfield Units
LM: Left main coronary artery
RCA: Right coronary artery
RMSE: Root-mean-square error
VOI: Volume-of-interest

## Declarations

### Guarantor

The scientific guarantors of this publication are Logan Hubbard and Sabee Molloi.

### Statistics and biometry

No complex statistical methods were necessary for this paper.

### Study subjects or cohorts overlap

Some study subjects or cohorts have been previously reported in: *Hubbard L, Malkasian S, Zhao Y, Abbona P, Molloi S (2019) Timing optimization of low-dose first-pass analysis dynamic CT myocardial perfusion measurment: validation in a swine model. European Radiology Experimental 3(16):1-9.*

### Methodology

- retrospective
- experimental
- performed at one institution
