# Supplementary material for: Contrast media timing optimization for coronary CT angiography: a retrospective validation study in swine
Source: Eur Radiol. 2022 Oct 11;33(3):1620–8. doi: 10.1007/s00330-022-09161-z (PMC9935703; doi:10.1007/s00330-022-09161-z)
Supplement: Supplementary file 1 — (DOCX 492 kb) [file 330_2022_9161_MOESM1_ESM.docx]

**Supplemental Material:**

**Supplemental Table 1: CCTA protocol timing, enhancement, and CNR comparisons versus the reference standard using the descending aorta for triggering**

| **PROTOCOL (N = 80)** | **PARAMETER**  **(MEAN ± SD)** | | **REFERENCE**  **(MEAN ± SD)** | **ERROR**  **(MEAN ± SD)** | **P-Value**  **(α< 0.05)** |
| --- | --- | --- | --- | --- | --- |
| **STANDARD CCTA (+5 seconds)** | | | | | |
| *Trigger-to-Acquisition Time (s)* | | 5.34 ± 0.21 | 6.92 ± 1.55 | -1.58 ± 1.52 | 0.00** |
| *Aortic Enhancement (HU)* | | 707.72 ± 202.08 | 759.89 ± 169.10 | -52.17 ± 82.41 | 0.20 |
| *LM Enhancement (HU)* | | 476.34 ± 192.90 | 515.46 ± 192.85 | -39.12 ± 103.40 | 0.27 |
| *RCA Enhancement (HU)* | | 441.40 ± 152.48 | 459.43 ± 175.33 | -18.03 ± 62.82 | 0.37 |
| *LM and RCA Enhancement (HU)* | | 458.87 ± 172.41 | 487.44 ± 183.99 | -28.57 ± 85.06 | 0.24 |
| *LM CNR* | | 13.20 ± 7.18 | 13.70 ± 7.17 | -0.49 ± 4.36 | 0.42 |
| *RCA CNR* | | 11.86 ± 6.02 | 11.50 ± 6.40 | 0.37 ± 2.88 | 0.43 |
| *LM and RCA CNR* | | 12.53 ± 6.57 | 12.60 ± 6.79 | -0.06 ± 3.67 | 0.48 |
| **OPTIMAL CCTA (+T_INJ_ / 2 + 1.5 seconds)** | | | | | |
| *Trigger-to-Acquisition Time (s)* | | 6.99 ± 1.36 | 6.92 ± 1.55 | 0.08 ± 0.72 | 0.32 |
| *Aortic Enhancement (HU)* | | 750.87 ± 158.31 | 759.88 ± 169.09 | -9.01 ± 23.92 | 0.43 |
| *LM Enhancement (HU)* | | 504.73 ± 192.43 | 515.46 ± 192.85 | -10.72 ± 28.20 | 0.43 |
| *RCA Enhancement (HU)* | | 437.01 ± 183.31 | 459.43 ± 175.33 | -22.42 ± 58.03 | 0.35 |
| *LM and RCA Enhancement (HU)* | | 470.87 ± 188.52 | 487.44 ± 183.99 | -16.57 ± 45.39 | 0.35 |
| *LM CNR* | | 13.85 ± 7.99 | 13.70 ± 7.17 | 0.16 ± 1.36 | 0.47 |
| *RCA CNR* | | 11.28 ± 7.31 | 11.50 ± 6.40 | -0.22 ± 2.05 | 0.46 |
| *LM and RCA CNR* | | 12.57 ± 7.67 | 12.60 ± 6.79 | -0.03 ± 1.72 | 0.49 |
|  | |  |  |  |  |

CCTA indicates coronary computed tomography angiography; N, number of measurements; HU, Hounsfield Units; STANDARD, standard CCTA protocol that uses a fixed delay of 5 seconds; OPTIMAL, optimal CCTA protocol that uses a variable delay of ½* Total Injection Time + 1.5 seconds; LM, left main coronary artery; RCA, right coronary artery; CNR, contrast-to-noise ratio. REFERENCE, corresponding result of the reference standard peak time or enhancement. **Indicates a p-value less than 0.05, i.e., significantly different from the reference standard

**
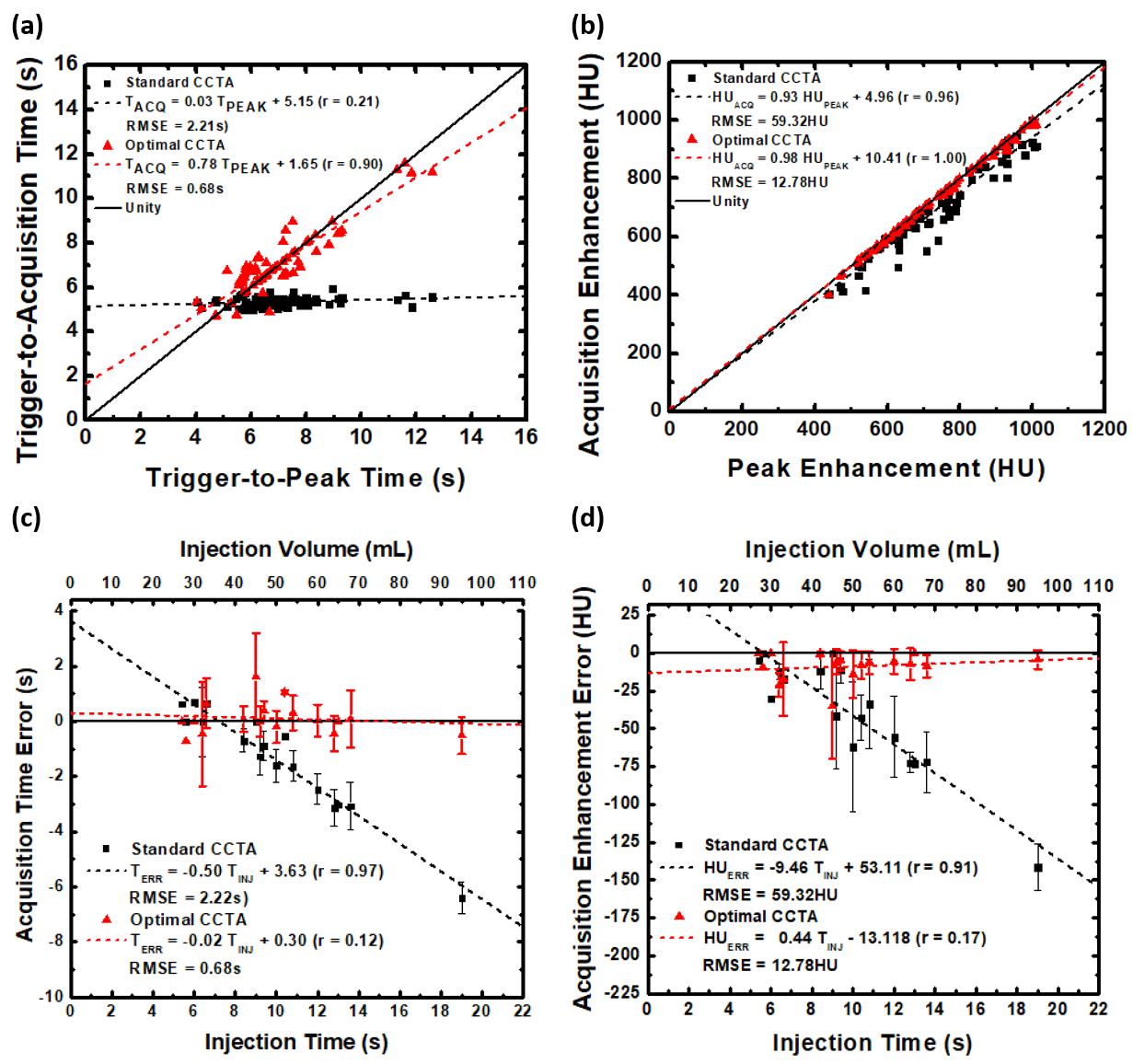
**

**Supplemental Figure 1. Acquisition timing and aortic enhancement of the standard and optimal CCTA protocols as compared to the reference standard when using the descending aorta for triggering**

**(a)** Protocol-dependent trigger-to-acquisition time versus the reference trigger-to-peak time. **(b)** Protocol-dependent aortic enhancement versus the reference peak aortic enhancement. **(c)** Protocol-dependent acquisition time error relative to the reference peak time as a function of injection time (or injection volume, as displayed by the double axis). **(d)** Protocol-dependent aortic enhancement error relative to the reference peak aortic enhancement as a function of injection time (or injection volume, as displayed by the double axis). In all cases, the standard CCTA protocol data is shown in black, with the optimal CCTA protocol data is shown in red. Importantly, for panels (c) and (d), clinically realistic injection times and volumes range from 10 or more seconds and 50-120 mL, respectively, at a rate of 5-7 mL/s[[15](#_ENREF_15)]. .
